# Elucidating respective functions of two domains BIR and C-helix of human IAP survivin for precise targeted regulating mitotic cycle, apoptosis and autophagy of cancer cells

**DOI:** 10.18632/oncotarget.22823

**Published:** 2017-12-01

**Authors:** Fabiao Hu, Daxia Pan, Wenyun Zheng, Ting Yan, Xiujuan He, Fuzheng Ren, Yiming Lu, Xingyuan Ma

**Affiliations:** ^1^ State Key Laboratory of Bioreactor Engineering, and School of Biotechnology, East China University of Science and Technology, Shanghai 200237, China; ^2^ Shanghai Key Laboratory of New Drug Design, and School of Pharmacy, East China University of Science and Technology, Shanghai 200237, China; ^3^ Department of Biochemical Pharmacy, School of Pharmacy, Second Military Medical University, Shanghai 200433, China

**Keywords:** BIR and CC domains, survivin, cell cycle, apoptosis and autophagy, breast cancer cells

## Abstract

Survivin was the smallest member of the IAP family, which was over expressed in many different cancers, and considered to be a promising hot target for cancer therapy, and our previous study demonstrated that multiple dominant negative mutants from full-length survivin could have many complex effects on cancer cells, such as cell cycle, apoptosis, and autophagy. But it was not yet known what role the two main domains played in those functions, which would be very important for the design of targeted anticancer drugs and for the interpretation of their molecular mechanisms. In this study, based on preparation the two parts (BIR domain and CC domain) of survivin by genetic engineering and cell characterization assay, we discovered that BIR (T34A)-domain peptide could inhibit Bcap-37 cells growth in a dose- and time-dependent manner, increase the proportion of G2/M phase, and induce caspase-dependent apoptosis via the mitochondrial pathway. While CC (T117A)-domain peptide increased the proportion of S-phase cells and increased the level of the autophagy marker protein LC3B significantly. These further experiments confirmed that TAT-BIR (T34A) peptide could be used to inhibit cell proliferation, promote apoptosis, and block mitosis, and TAT-CC (T117A) peptide showed mainly to promote autophagy, process of DNA replication, and mitosis to breast cancer cells. This research will lay the foundation for interpreting the multifunction mechanism of survivin in cell fates, further make senses in developing the anticancer drugs targeting it precisely and efficiently.

## INTRODUCTION

**Figure 1:**
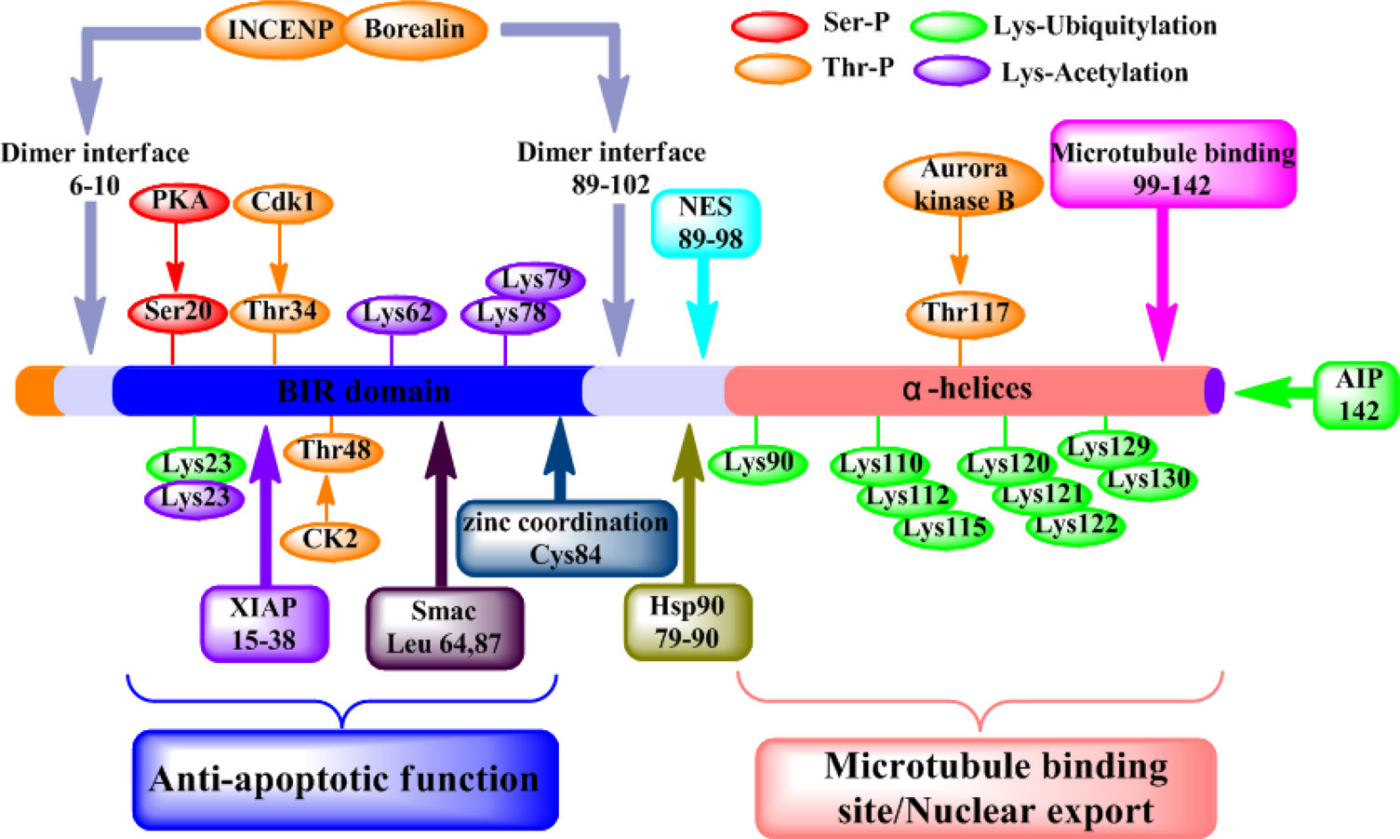
Structure and function of survivin protein Various functional motifs in survivin are indicated, including the binding sites for protein partners, dimer interface (residues 6-10 and 89-102), XIAP (residues 15-38), Smac (residues 64 and 87), Hsp90 (residues 79-90), nuclear export sequence (NES) (residues 89-98), polymerized microtubules (residues 99-142) and AIP (residue 142). And the position of experimentally validated phosphorylation sites for PKA (Ser20) p34cdc2/Cdk1 (Thr34) and Aurora B (Thr117). Residues involved in the Zn^2+^ coordination sphere (Cys84), Ser/Thr phosphorylation, acetylation or ubiquitylation.

Survivin is the smallest member of the inhibitor of apoptosis (IAP) family, which is overexpressed in most of human tumors but not in normal tissues [1, 2]. Overexpression of survivin not only plays a key role in the regulation of apoptosis and cell division [3], but also correlates with tumor progression and induces anticancer drug resistance [4, 5]. Thus, it has become a potential oncotherapeutic target. Unlike other family members of IAP, survivin contains 142 amino acids and folds into two significant domains: a baculoviral IAP repeat (BIR) domain in the N-terminus (100aa) and an elongated α-helical coiled-coil (CC) in the C-terminal (42aa) [6, 7] (Figure 1). X-ray crystallography data has shown two molecules of survivin can form a bowtie-shape dimer through a hydrophobic interface [8], which are formed by the 6-10 residues immediately prior to the BIR domain region at N-terminal and the 14 residues (survivin 89-102) in the connecting region between the BIR domain and the C-terminal α-helix structure [9]. Moreover, the differential functions of survivin seem to be caused by differential subcellular localization of this molecule [10]. Nuclear localization of survivin is mainly involved in spindle monitoring at mitosis, whereas cytoplasmic/mitochondrial survivin counteracts pro-apoptotic signals by preventing caspase-9 and caspase-3 activation [11].

During mitosis, survivin is localized to various components of the mitotic apparatus, including centrosomes, microtubules of the metaphase and anaphase spindle, and the remnants of the mitotic apparatus-midbodies [12]. A direct association between survivin and polymerized tubulin has been demonstrated *in vitro* [13], potentially indicating the involvement of the C-terminal α-helices. Meanwhile, survivin can also promote mitosis by forming the chromosomal passenger complex (CPC) with Aurora-B kinase, inner centromere protein (INCENP), and Borealin [14]. The CPC facilitates the correction of maloriented chromosomes during prometaphase congression and the execution of cytokinesis [15, 16]. Phosphorylation at threonine 117 of survivin by Aurora-B kinase was reported to regulate the entire chromosomal passenger complex in mammals [17]. Wheatley et al. [18] reported that, the non-phosphorylatable survivin (survivinT117A) could substitute for the wild type protein, while the phosphomimic (survivinT117E) could not restore viability, nor could it complement chromosome congression and spindle checkpoint defects that arose due to depletion of endogenous survivin.

Overexpression of survivin has been associated with inhibition of cell death initiated via the extrinsic or intrinsic apoptotic pathways [3]. Survivin interferes with the process of apoptosis through inhibition of different caspase activity by the interaction between the single BIR domain of survivin and different caspases [19]. *In vitro* study indicates that Thr34 phosphorylation of survivin by CDC2 is essential for the interaction of survivin with caspase-3, 7 and 9 [20]. Further studies have found that a mutation of Survivin(T34A) can induce the release of cytochrome c from the mitochondria, leading to apoptosis [21]. According to these studies, we discovered that single BIR domain of survivin interfered with the process of apoptosis through inhibition of different caspase activity. Therefore, we urgent to know that whether CC domain of survivin interferes with the process of apoptosis. In addition, autophagy is closely linked with apoptosis by shared regulatory systems and common pathways, indicating its relevance with apoptosis and important role in tumorigenesis [22]. Beclin-1 can positively regulate autophagy by combining with PI3KCIII/Vps34 and other positive co-factors such as Survivin, Akt, and Bcl-2/Bcl-X_L_ to form the Beclin-1 interactome [23]. Recent study indicates that interaction of Beclin-1 with survivin regulates sensitivity of human glioma cells to TRAIL (a death receptor ligand)-induced apoptosis [24]. Ectopic expression of survivin also significantly attenuated YM155-induced apoptosis and autophagy, whereas survivin siRNA induced autophagy [25]. Chang et al. [26] demonstrated that silencing survivin slightly influenced cell growth in HCC cells and induced the formation of autophagosomes. These literatures only explained that up-regulation of survivin inhibited autophagy, while down-regulation of survivin promoted autophagy. However, the mechanism of survivin regulating autophagy has not been resolved. For that reason, we speculated that whether BIR domain of survivin of cancer cells inhibited autophagy by inhibiting apoptosis, or CC domain of survivin also interfered autophagy.

Our previous studies demonstrated that multiple dominant negative mutants from full-length survivin could cause cancer cells to have many complex effects such as cell cycle, apoptosis, and autophagy [27, 28]. However, the role(s) and mechanisms that each domain may play in regulating the cell cycle, autophagy, and apoptosis, have not been reported. In this study, we separately prepared the two individual domains (BIR domain and CC domain) as the truncated versions of survivin (namely TAT-BIR(T34A) and TAT-CC(T117A) ) and systematically explored the functions of them in the cell cycle, apoptosis, and autophagy of breast cancer. We found that TAT-BIR (T34A) could be used to inhibit cell proliferation, promote apoptosis, and block mitosis, and TAT-CC (T117A) peptide showed mainly to promote autophagy, process of DNA replication, and mitosis to breast cancer cells.

## RESULTS

### TAT-BIR(T34A) can inhibit growth of cultured breast cancer cell lines

**Figure 2:**
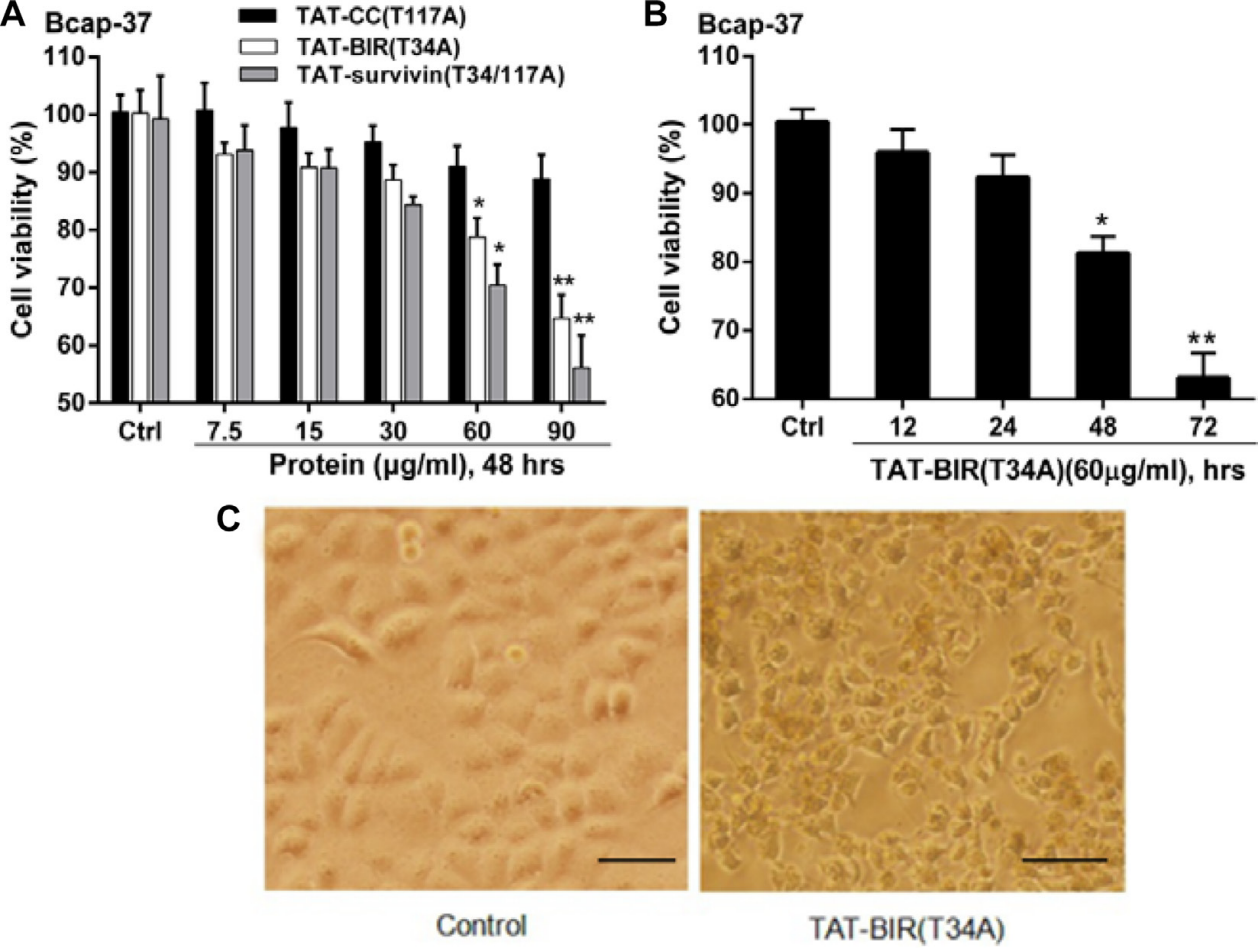
TAT-BIR(T34A) inhibit proliferation in Bcap-37 cells (**A**) Viability of Bcap-37 cells after treated with recombinant proteins (TAT-Survivin(T34/117A), TAT-BIR(T34A), and TAT-CC(T117A)). Bcap-37 cells were exposed to recombinant proteins at a various concentrations (7.5, 15, 30, 60, and 90 µg/mL) for 48 h; cell viability was measured by MTT assay. The data presented was means ± SD of 3 independent experiments. ^*^P < 0.05 and ^**^P < 0.01 compared with the control group. (**B**) Viability of Bcap-37 cells treated with 60 µg/mL TAT-BIR(T34A) for different time (12, 24, 48 and 72 hours). The cell viability was measured by MTT assay. Data represented was the means ± SD of three independent experiments. ^*^P < 0.05 and ^**^P < 0.01 compared with the control group. (**C**) The morphological change of Bcap-37 cells treated with 60 µg/mL TAT-BIR(T34A) for 24 h. (Ba r = 50 µm)

To establish whether these truncated versions of survivin could inhibit cell growth, the cell-inhibitory effect of TAT-BIR(T34A), TAT-CC(T117A), and TAT-Survivin(T34/117A) were determined by MTT assay on human breast carcinoma cell lines Bcap-37. After treated with a various concentrations of TAT-CC(T117A), TAT-BIR(T34A), and TAT-Survivin(T34/117A) (7.5, 15, 30, 60, and 90 µg/mL, respectively), the viability of Bcap-37 cells decreased in a dose-dependent manner (Figure 2A). The cell viability of Bcap-37 cells treated with TAT-CC(T117A) decreased from 100% to 90.2%, TAT-BIR(T34A) from 100% to 64.6%, and TAT-Survivin(T34/117A) from 100% to 56.1%. These data demonstrated that TAT-BIR(T34A), the single domain of survivin, still could inhibit cell growth, and showed anticancer effect similar to that of the full-length TAT-Survivin(T34/117A), while TAT-CC(T117A) had little effect on cell proliferation. Specifically, the cell viability of Bcap-37 cells incubated with 60 µg/mL TAT-BIR(T34A) exhibited a gradually decline for 12, 24, 48 and 72 h, respectively (Figure 2B). Meanwhile, we witnessed the remarkable morphological changes that embody early apoptosis of treated cells, such as irregular shape, shrinkage, and the rough edge (Figure 2C). Therefore, the anti-proliferation activity of TAT-BIR(T34A) showed the dose- and time-dependent.

**Figure 3:**
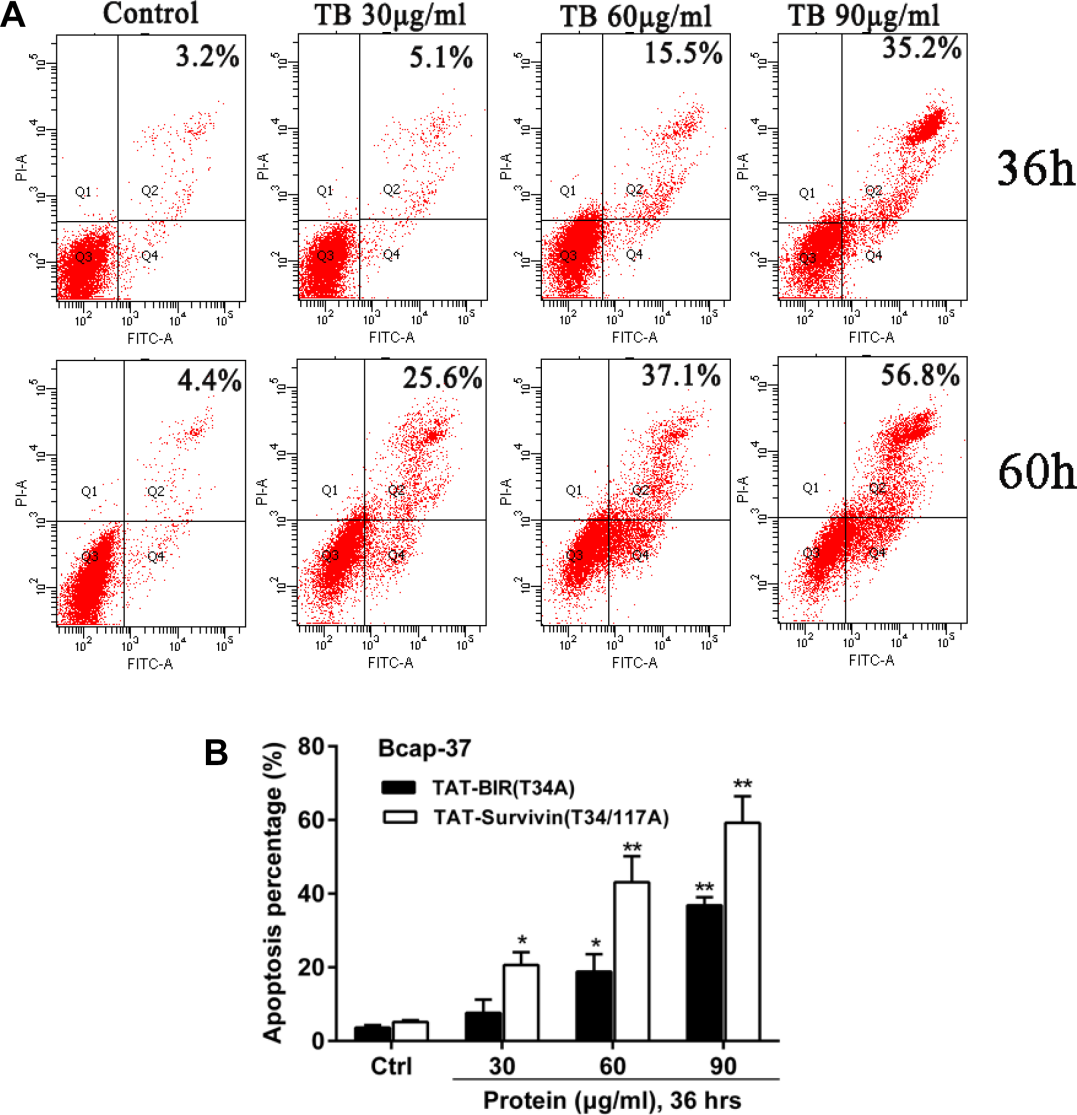
Apoptosis of Bcap-37 cells was analyzed by flow cytometry (**A**) Apoptosis assay of Bcap-37 incubated with different concentration (30, 60, and 90 µg/mL) of TAT-BIR(T34A) for 36 h or 60 h. (**B**) Apoptosis analysis of cells treated with different concentration (30, 60, and 90 µg/mL) of TAT-BIR(T34A) or TAT-Survivin(T34/117A) for 36 h. The apoptosis percentage was the means ± SD of 3 independent experiments. ^*^P < 0.05 and ^**^P < 0.01 compared with the control group.

Annexin V-FITC/PI experiment was carried out to further determined the effect of TAT-BIR(T34A) and TAT-Survivin(T34/117A) on cell apoptosis. After treated with different concentrations (30, 60, and 90 µg/mL) of TAT-BIR(T34A), the apoptosis rate of Bcap-37 cells increased gradually (Figure 3A). Apoptosis rate was 3.2% and 35.2% when incubated with 30 µg/mL and 90 µg/mL TAT-BIR(T34A) for 36 h, respectively. When the treated time was 60 h, it changed to 4.4% and 56.8%, showing an approximately dose- and time-dependent. These above results suggested that BIR(T34A) domain could promote apoptosis of Bcap-37 cells. Moreover, it was also found that cell apoptosis in TAT-Survivin(T34/117A) group was higher than that in TAT-BIR (T34A) group (Figure 3B).

**Figure 4:**
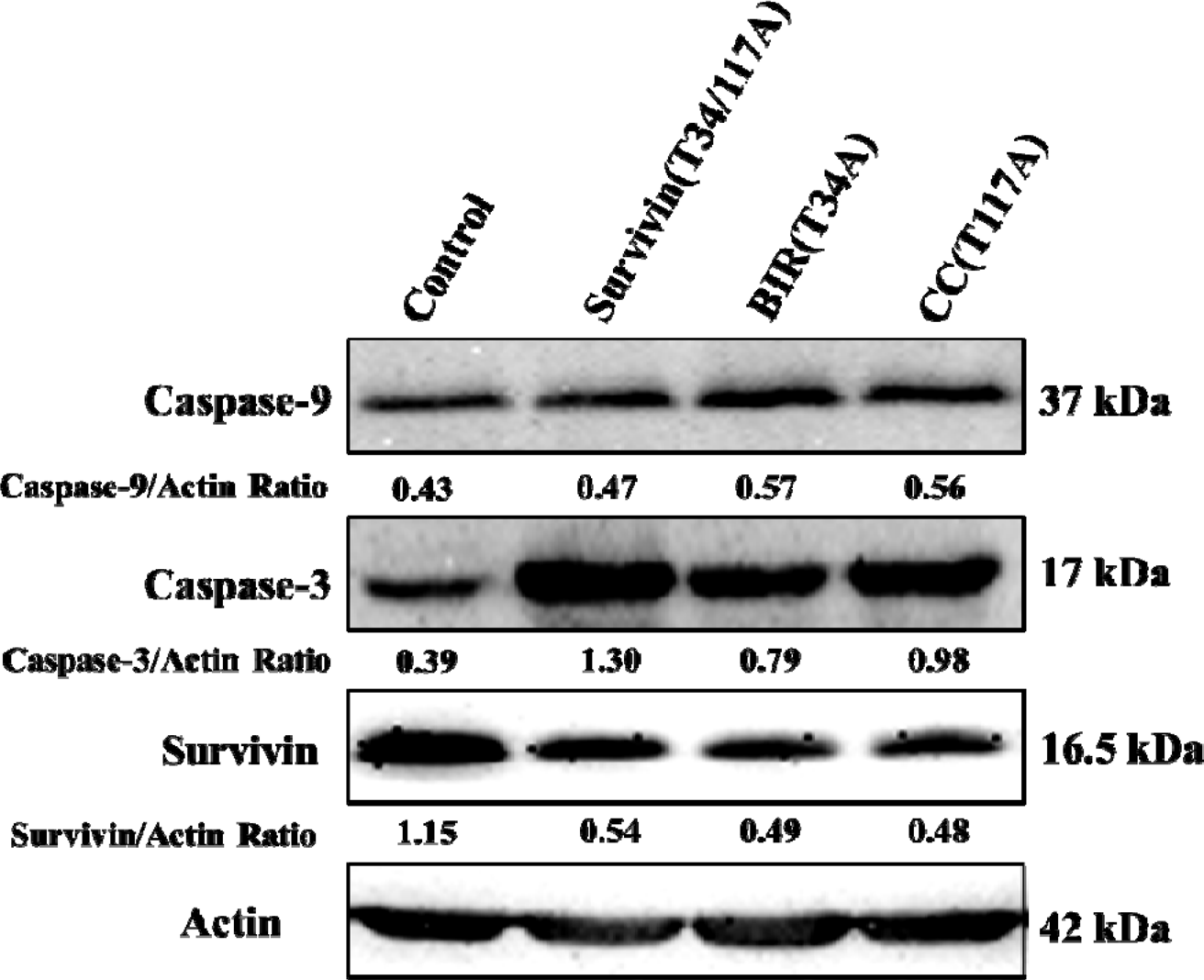
Western blot analysis of apoptosis-related proteins Activated caspase-9, activated caspase-3, and survivin were detected by western blot with cellular extracts of Bcap-37 cells treated with each of the three kinds of proteins for 48 h. Actin was used to confirm equal protein loading on the gel and to show the relative purity. The grey value of protein bands have been quantified by Image J.

To confirm the main apoptotic pathway of Bcap-37 cells induced by TAT-BIR(T34A), TAT-CC(T117A), and TAT-Survivin(T34/117A), the expression of apoptosis-associated proteins, activated caspase-9, activated caspase-3 and survivin, were analyzed by Western blot. Compared with the control group, the level of survivin descended significantly and activated caspase-3 increased in the Bcap-37 cells treated with each of the three proteins for 48 h. However, the level of activated caspase-9 increased slightly. It was noteworthy that the treatment of TAT-Survivin(T34/117A) resulted in a more significant increase of activated caspase-3 (Figure 4). The above mentioned results indicated that, pro-apoptosis associated proteins activated caspase-9 and caspase-3 increased, whereas anti-apoptosis associated proteins survivin decreased in Bcap-37 cells treated with each of the three proteins.

### Effects of TAT-BIR(T34A), TAT-CC(T117A) , and TAT-Survivin(T34/117A) on cell cycle of Bcap-37 cells

**Figure 5:**
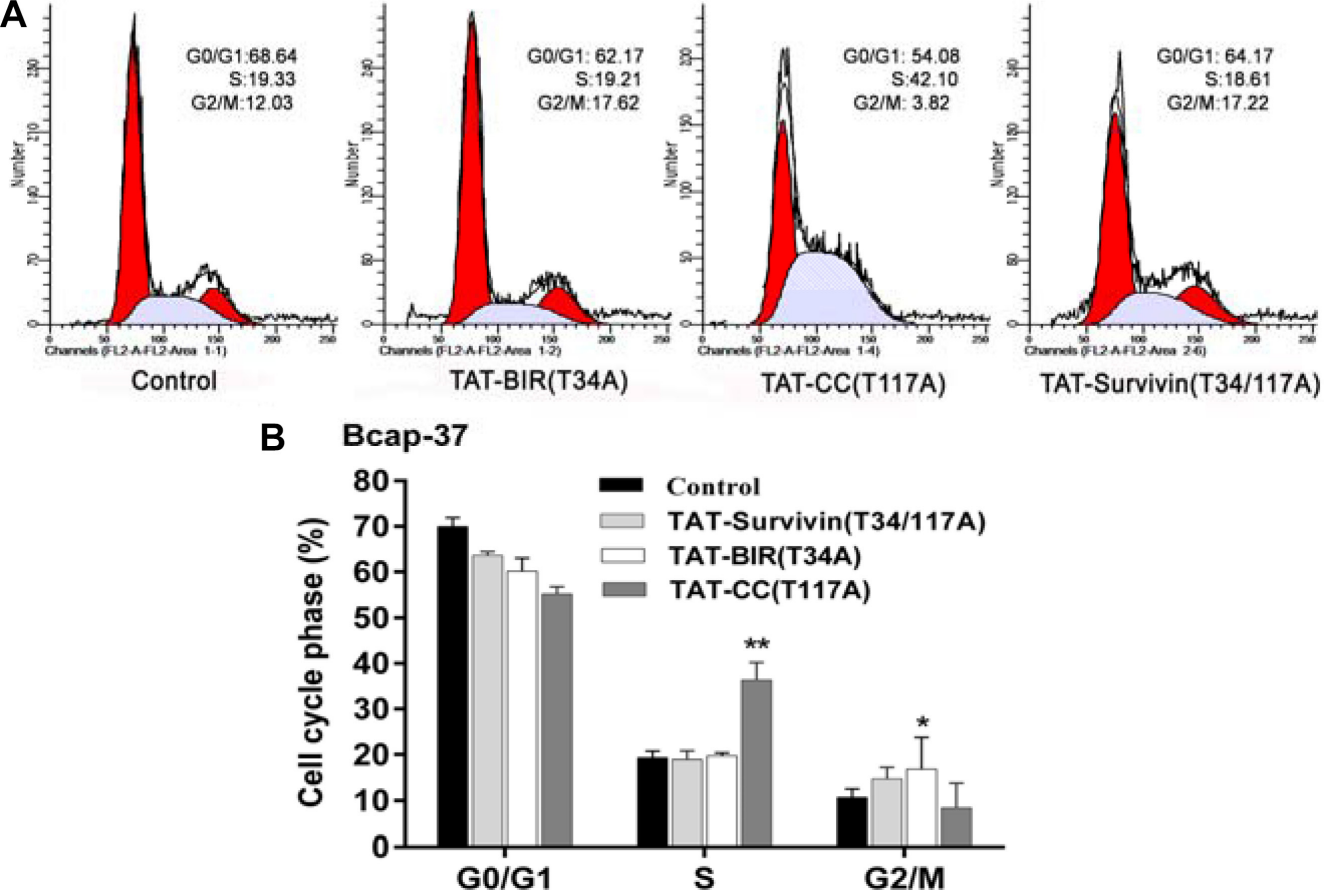
Cell cycle distribution of Bcap-37 treated with different proteins Bcap-37 cells were treated with 30 µg/mL TAT-BIR(T34A), TAT-CC(T117A), and TAT-Survivin(T34/117A) for 48 h. The cells were fixed by ice-cold 70% (v/v) ethanol, stained with 50 µg/mL PI, and analyzed by flow cytometry. (**A**) Cell cycle distribution analyzed by flow cytometry. (**B**) Histogram of cell cycle distribution of Bcap-37 cells incubated with three kinds of protein. The data presented are the means ± SD of 3 independent experiments. ^*^P < 0.05 and ^**^P < 0.01 compared with the control group.

To determine the function of these proteins in the regulation of cell cycle, the distribution of cell cycle in Bcap-37 cells treated with TAT-BIR(T34A), TAT-CC(T117A), and TAT-Survivin(T34/117A) was analyzed by flow cytometry. In comparison with the control group, cell proportion of G0/G1 phase all declined in the treated group, and cell proportion of G2/M phase and S phase showed the different change in Bcap-37 cells. The cell proportion of G0/G1 phase in TAT-BIR(T34A)- and TAT-Survivin(T34/117A)-treated group exhibited a slight decrease, which were 6.47% and 4.27%, respectively. However, the cell proportion of G2/M phase in TAT-BIR(T34A)- and TAT-Survivin(T34/117A)-treated group both increased by 5%. It was surprised that TAT-CC(T117A) treatment led to the proportion of cells in G0/G1 phase decreased significantly by 14.6%, and the proportion of cells in S phase increased by 22.17%, from 19.33% to 42.10% (Figure 5A). All the evidences suggested that TAT-BIR(T34A) and TAT-Survivin(T34/117A) treatment arrested cell cycle at G2/M phase in Bcap-37 cells, and showed the similar effects on the distribution of the cell cycle. However, TAT-CC(T117A) displayed the different functions, which enabled proportion of cells in G0/G1 phase fell sharply, and proportion of cells in S phase increased significantly (Figure 5B). It demonstrated that the CC domain promoted the Bcap-37 cells to leave the G0/G1 resting phase and to enter the stage of DNA replication, and thus promoting cell division.

**Figure 6:**
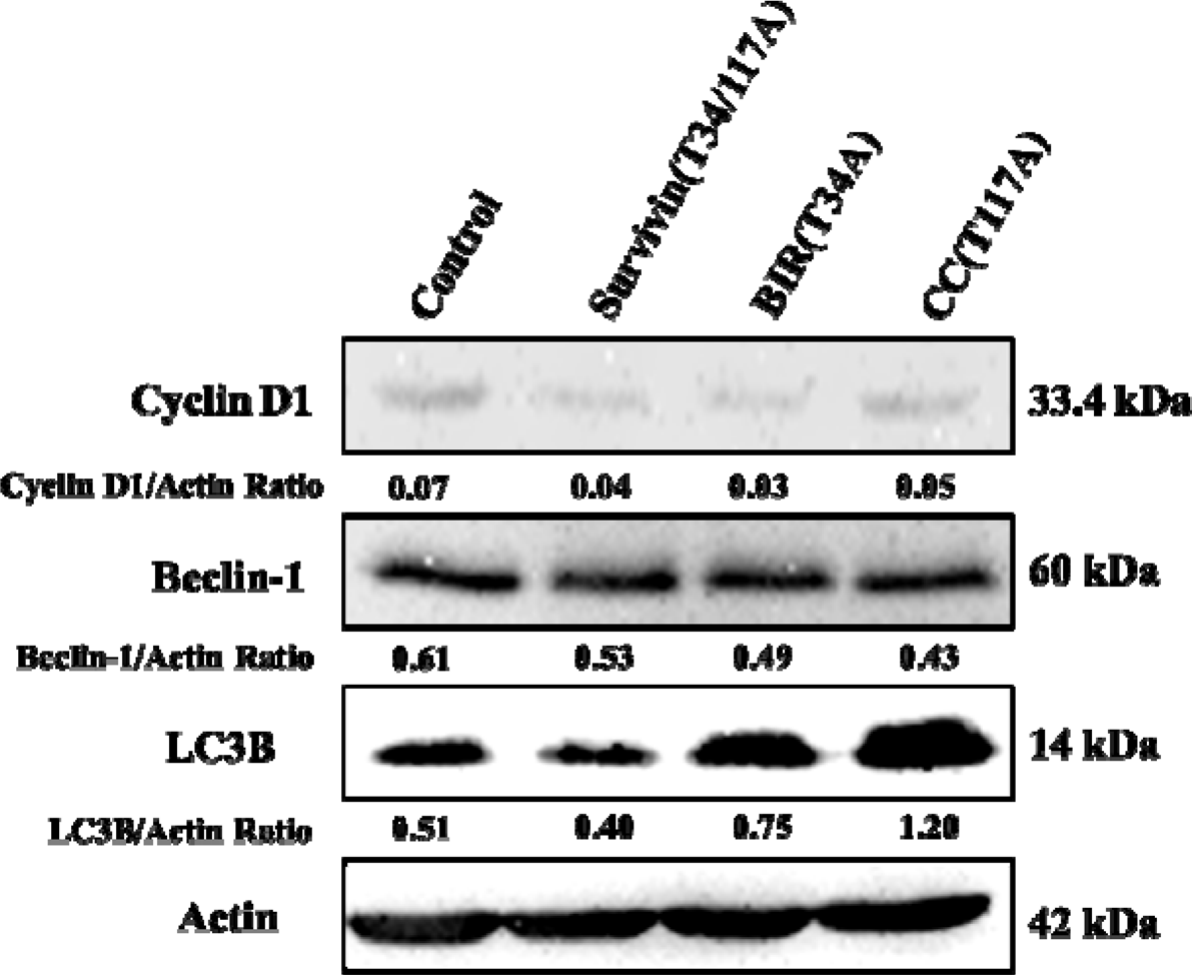
Western blot analysis for the activation of autophagy- and cell cycle-related proteins The Bcap-37 cells were incubated with three kinds of recombinant proteins for 48 h, and their cellular extracts were detected by western blot for Cyclin D1, Beclin-1, and LC3B. Actin was used to indicate comparable amount of protein loading on the gel. The grey value of protein bands have been quantified by Image J.

Cyclin D1 is a protein that regulates cell cycle, which is first synthesized in the cell cycle and peaked at mid-G1 phase [29]. Hence, the expression of Cyclin D1 was determined by Western blot. The results illustrated that the expression of Cyclin D1 decreased in Bcap-37 cells treated with three recombinant proteins (Figure 6), namely that the proportion of cells in G1 phase decreased resulted from Cyclin D1 decreased. However, the Cyclin D1 level of TAT-CC(T117A)-treated group was higher than that in TAT-BIR(T34A) group and TAT-Survivin(T34/117A) group, suggesting TAT-CC(T117A) could promote Bcap-37 cells to leave the G0/G1 resting phase, and enter the stage of DNA replication. These results were consistent with the observations in flow cytometry analysis lowered proportion of cells in G0/G1 of treated Bcap-37.

### Immunofluorescence assay of mitochondrial membrane potential in Bcap-37 cells

**Figure 7:**
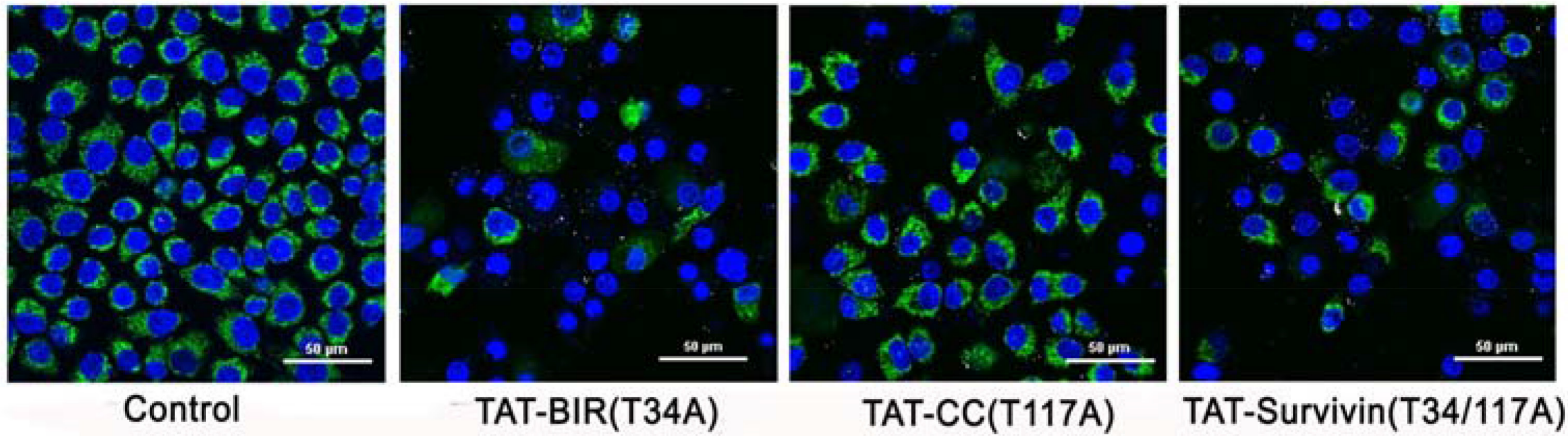
The change of mitochondrial membrane potential of Bcap-37 cells The Bcap-37 cells after incubated with TAT-BIR(T34A), TAT-CC(T117A), and TAT-Survivin(T34/117A) for 48 h were placed in confocal laser scanning microscopy to observe cell fluorescence. The change of mitochondrial membrane potential was detected using Rhodamine 123 and Hoechst33342 staining. Rhodamine 123 could enter mitochondrial matrix and appeared green, while Hoechst33342 would stain mitochondrial DNA blue. The intensity of green fluorescence illustrated the membrane potential. (Bar = 50 µm)

The loss of mitochondrial membrane potential was one of the typical characteristics of apoptosis [30]. In this study, the change of mitochondrial membrane potential in Bcap-37 cells treated with TAT-Survivin(T34/117A), TAT-BIR(T34A), and TAT-CC(T117A) for 48 h was detected using Rhodamine 123 and Hoechst33342 staining. The intensity of green fluorescence in TAT-BIR(T34A)- and TAT-Survivin(T34/117A)-treated group decreased drastically compared with that of control group, and even no green fluorescence was seen in some cells. However, the intensity of green fluorescence in TAT-CC(T117A)-treated group was not significant (Figure 7). These phenomena suggested that the mitochondria was not stained by Rhodamine 123 due to lowered mitochondrial membrane potential in Bcap-37 cells treated with TAT-BIR(T34A) and TAT-Survivin(T34/117A). In other words, TAT-BIR(T34A) promoted the apoptosis of Bcap-37 cells by interfering the membrane potential of the mitochondria, and further the normal functions of mitochondria.

### Immunofluorescence for localization of recombinant proteins in Bcap-37 cells

**Figure 8:**
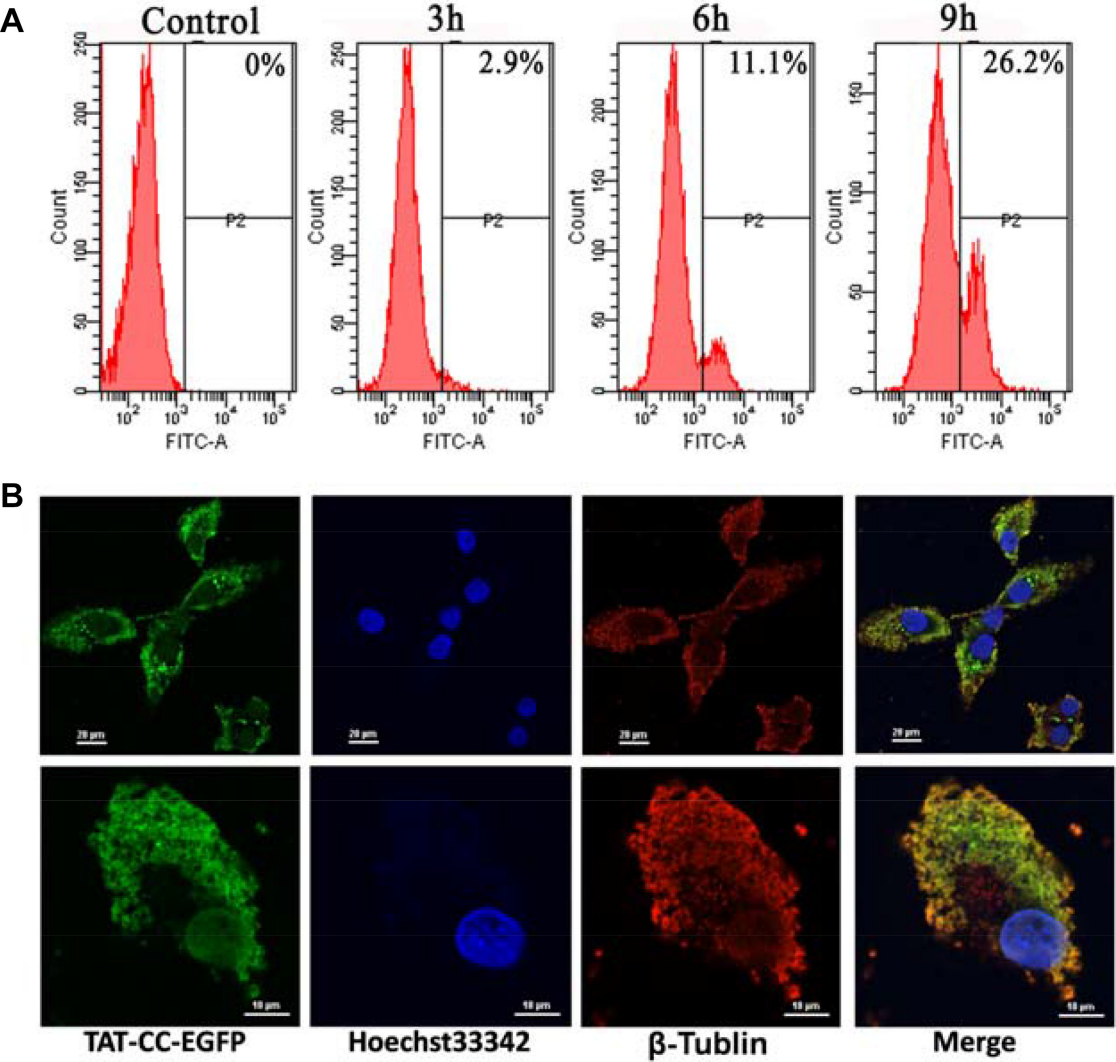
Cellular entry and location of TAT-CC-EGFP (**A**) Cellular entry of TAT-CC-EGFP determined by flow cytometry. The cell was treated with TAT-CC-EGFP for different time points (3 h, 6 h, and 9 h), and analyzed by flow cytometry. (**B**) The localization of TAT-CC-EGFP in Bcap-37 cells. Bcap-37 cells were incubated with TAT-CC-EGFP for 48 h, fixed by 4% (v/v) paraformaldehyde (PA), then treated by 0.1% (v/v) Triton X-100, and blocked for 1 h at room temperature with 3% BSA. Tubulin was probed for monoclonal antibody against tubulin followed by cy3-conjugated goat anti-rabbit secondary antibody. TAT-CC-EGFP entered cells and appeared green. DNA dyed by Hoechst33342 was blue. Tubulin was red. (Up, bar = 20 µm; down, bar = 10 µm)

In order to observe the localization of recombinant proteins, Bcap-37 cells were treated with enhanced green fluorescent protein (EGFP) conjugated TAT-CC(T117A) (namely TAT-CC-EGFP) for 48 h. We firstly assayed the penetrating ability of recombinant proteins with TAT peptide into cells by detecting the proportion of fluorescent protein with flow cytometry. The cells with green fluorescence accounted for 2.9%, 11.1%, and 26.2%, after incubated with TAT-CC-EGFP for different period of time (3 h, 6 h, and 9 h) (Figure 8A), respectively. It exhibited that TAT-CC(T117A) protein had a higher penetrating efficiency, the result was consistent with Cho et al. [31]. Furthermore, TAT-CC-EGFP was able to enter cells, abounded in the cells, and appeared green, whereas tubulin was red, and superimposed with green (Figure 8B). These results indicated that CC domain of survivin could bind with tubulin, and localize in microtubules, suggesting CC domain of survivin might associate with microtubules of the mitotic spindle at the beginning of mitosis, and regulate microtubule dynamics.

### Autophagy of Bcap-37 cells treated with recombinant proteins

**Figure 9:**
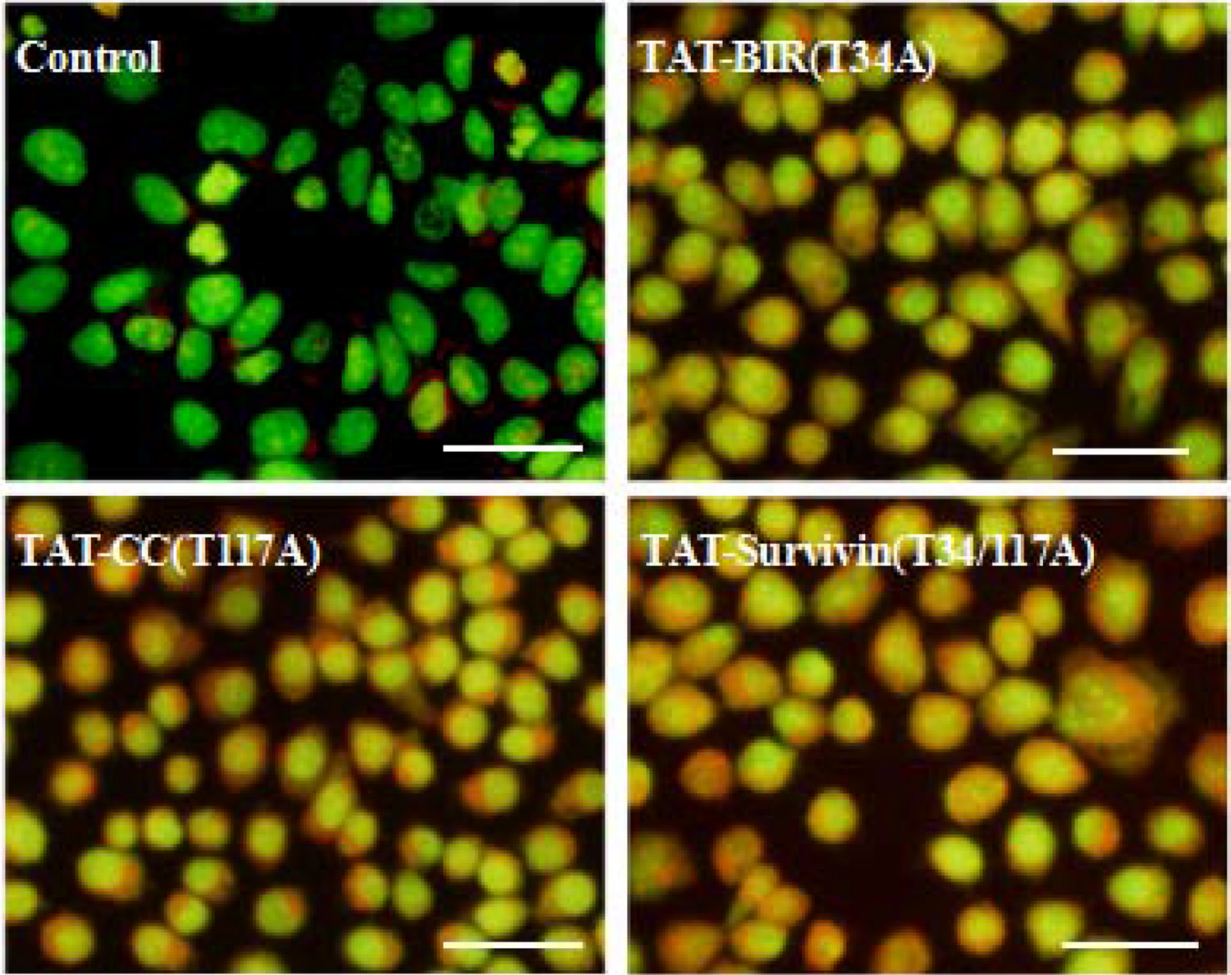
Detection of autophagy of treated Bcap-37 cells using acridine orange staining Bcap-37 cells (2 × 10^4^ cells/well) were seeded into 6-well plate overnight, and then treated with TAT-BIR(T34A), TAT-CC(T117A), and TAT-Survivin(T34/117A) for 48 h. The harvested cells were stained with acridine orange, and the fluorescence was observed with microscopy. (Bar = 50 µm)

Acridine orange was a kind of cell-permeable fluorescent dye, and dyed cytoplasm and DNA bright green [32]. Meanwhile, it could penetrate into acidic organelles, such as autophagy-lysosome, and showed red fluorescence in lower pH, and the intensity was related to the degree of acidity [33]. After stained with acridine orange, cell nucleus of the control group appeared green, suggesting there was no detective acidic organelles, such as autophagy-lysosome. However, the intensity of red fluorescence increased in the cytoplasm of Bcap-37 cells treated with TAT-Survivin(T34/117A), TAT-BIR(T34A), and TAT-CC(T117A) for 48h, and emerged yellow after superimposed green (Figure 9), indicating acidic organelles increased in the treated group. It was noteworthy that intracellular acidic organelles increased more in the Bcap-37 cells treated with TAT-CC(T117A) and TAT-Survivin(T34/117A) than that in the group treated with TAT-BIR(T34A). It also implied that TAT-CC(T117A) might promote autophagy.

**Figure 10:**
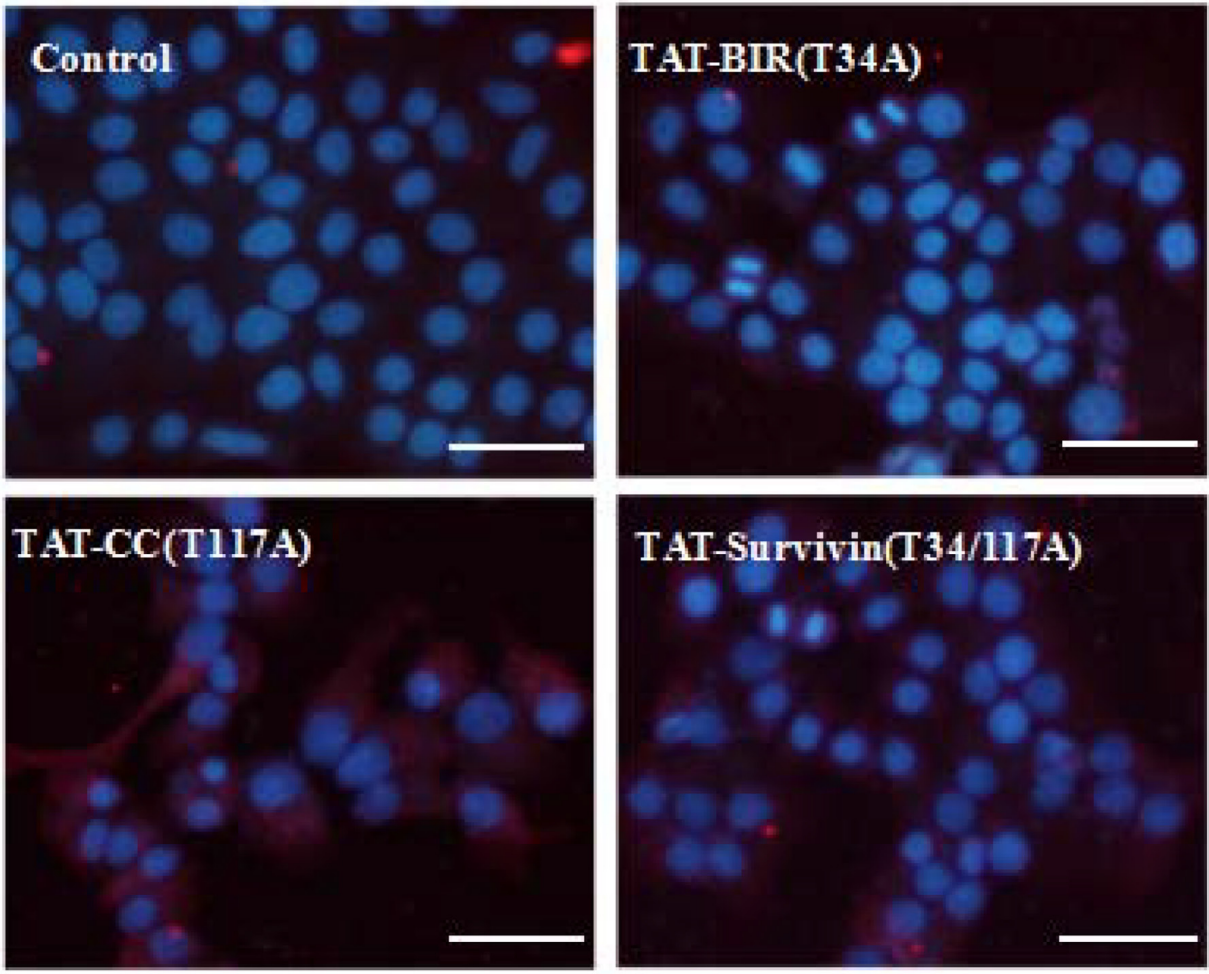
Detection of LC3B in treated Bcap-37 cells using immunofluorescence assay After incubated with TAT-BIR(T34A), TAT-CC(T117A), and TAT-Survivin(T34/117A) for 48 h, LC3B protein of Bcap-37 cells was detected with LC3B antibody which displayed cy3-marked fluorescent secondary antibodies, showing red. Nuclear DNA dyed by Hoechst33342 was blue. (Bar = 50 µm).

At the same time, in this study, LC3B protein, one of the light chain 3(LC3) members characterizing the autophagy, was detected with LC3B antibody in Bcap-37 cells incubated with TAT-BIR(T34A), TAT-CC(T117A), and TAT-Survivin(T34/117A) for 48 h. The level of LC3B protein increased slightly in Bcap-37 cells treated with TAT-BIR(T34A). However, it increased significantly in Bcap-37 cells incubated with TAT-CC(T117A) and TAT-Survivin(T34/117A) (Figure 10). Western blot identified that the expression of LC3B in TAT-CC(T117A)-treated group was highest, and was about 2.35-fold of that in the control group by quantification (Figure 6). It was exciting that TAT-CC(T117A) treatment leaded to the highest level of autophagy of Bcap-37 cells, which was consistent with the result of immunofluorescence, indicating TAT-CC(T117A) could promote autophagy. Moreover, Beclin-1 was the only tumor suppressor gene that had been confirmed to associate with autophagy in mammals [34]. Western blot evaluated that the expression of Beclin-1 in TAT-BIR(T34A)-, TAT-CC(T117A)-, and TAT-Survivin(T34/117A)-treated group was slightly lower than the control group, while the expression of Beclin-l in TAT-CC(T117A)-treated group was higher than TAT-BIR(T34A)- and TAT-Survivin(T34/117A)-treated group (Figure 6), suggesting TAT-CC(T117A) could promote autophagy by increased the level of Beclin-1.

## DISCUSSION

The multifunction of survivin in apoptosis, cell cycle, and autophagy had been reported in recent years [35, 36]. However, the specific function of two different domains of survivin in these functions was unclear. After separated two structures (BIR domain and CC domain) of full-length survivin, our results confirmed that a single BIR domain (T34A) structure still had the ability to inhibit proliferation of cancer cell, while CC domain (T117A) had little effect on cell proliferation (Figure 2A). Moreover, TAT-BIR(T34A) could inhibit growth of Bcap-37 cells and induce apoptosis in a dose- and time-dependent manner (Figure 2 and Figure 3A). The difference on cell proliferation and apoptosis implied that the pro-apoptotic domain mutant of survivin existed in BIR domain mutant (BIR(T34A)) rather than CC domain mutant (CC(T117A)). Our previous studies also found that TAT-Survivin(T34A) inhibited proliferation of Bcap-37 cells, whereas TAT-Survivin(T117A) had little effect on cell proliferation [27]. As previously reported, because its BIR motif, survivin was a potential member of the inhibitor of apoptosis (IAP) family of proteins, which acted at discrete steps to regulate the apoptotic pathway of cell death [3]. Phosphorylation at threonine 34, within its BIR domain, by Cdk1 was critical to anti-apoptotic roles of survivin. Further studies have found that the non-phosphorylatable form, survivinT34A, accelerated cell proliferation and promoted apoptosis, whereas survivinT34E retarded growth and promoted survival [37]. Surprisingly, we observed that the effect of pro-apoptosis of single BIR(T34A) was much weaker than full-length Survivin(T34/117A) (Figure 3B). As previously described, dimer interface included the 6-10 residues of N-terminal and the 14 residues (survivin 89-102) in the connecting region between the BIR domain and the C-terminal α-helix structure [9]. Because TAT-Survivin(T34/117A) possessed complete amino acids of dimer interface compared to TAT-BIR(T34A), TAT-Survivin(T34/117A) bound more wild-type survivin than TAT-BIR(T34A), leading to higher apoptosis rate of Bcap-37 cells. Moreover, the effect of pro-apoptosis of full-length Survivin(T34/117A) was much stronger than full-length Survivin(T34A) [27], which also eliminated the effect of the CC domain on apoptosis. There are two main pathways of apoptosis in mammals, namely endogenous (mitochondrial pathway) and exogenous pathways [3]. In order to further determine the main apoptotic pathway of Bcap-37 cells induced by BIR domain mutant, the expression of apoptosis-associated proteins and mitochondrial membrane potential were analyzed by western blot and immunofluorescence. Western blot discovered that pro-apoptosis associated proteins activated caspase-9 and caspase-3 increased, whereas anti-apoptosis associated proteins survivin decreased (Figure 4). Survivin inhibited activation of procaspase-9 by interfering with the apoptosome formation. Active caspase-9, however, activated effector caspases-3 and caspases-7, which in turn induced apoptosis [21]. *In vitro* study indicated that Thr34 phosphorylation of survivin by CDC2 was essential for the interaction of survivin with caspase-3, 7 and 9 [20]. It demonstrated that single BIR(T34A) was heterologous to wild-type survivin, which resulted in the ubiquitin-dependent degradation of survivin, prevented wild-type survivin from interacting directly with caspase-3, and prompted survivin decreased and caspase-3 increased. Mitochondrial dysfunction was often related to the loss of mitochondrial membrane potential (MMP) and the release of cytochrome [38]. Our studies ascertained that TAT-BIR(T34A) promoted the apoptosis of Bcap-37 cells by interfering the membrane potential of mitochondria. Some studies have found that a mutation of Survivin(T34A) could induce the release of cytochrome c from the mitochondria, and caused apoptosis [21]. For that reason, these data suggested that BIR domain mutant (BIR(T34A)) inhibited cell proliferation and induced caspase-dependent apoptosis via the mitochondrial pathway.

Survivin mRNA expression is regulated by the cell cycle, and it peaks in the G2/M phase and rapidly declines in the G1 phase [39]. Furthermore, overexpression of survivin can accelerate S phase shift through a mechanism involving interaction with CDK4, counteract G1 arrest, and result in phosphorylation of retinoblastoma protein (Rb), a tumor suppressor protein [40]. Barrett et al. [37] discovered that the cell cycle profile of T34A-treated cells was normal, whereas T34E-treated cells had a significantly increased G2/M population, suggesting that phosphorylated Thr34 inhibits mitosis. On the contrary, our previous experiments found that non-phosphorylatable survivin mutants Survivin(T34A) and Survivin(T34/117A) arrested cell in G2/M phase. Based on this contradiction, we discovered TAT-BIR(T34A) abolished kinase p34^cdc2^-cyclin B1 on the survivin phosphorylation, and disrupted the biological function of wild-type survivin, leading to obvious cell cycle arrested at G2/M phase (Figure 5A and B), which might cause failure of cell division. In addition, survivin could also promote mitosis by forming the chromosomal passenger complex (CPC) with Aurora-B kinase, inner centromere protein (INCENP), and Borealin [41]. It localized to the spindle microtubule organizing center (MTOC) during the G2/M phase of cell cycle and regulated microtubule dynamics by a direct association with polymerized tubulin [14]. Meanwhile, survivin was phosphorylated by Aurora-B kinase at threonine 117 in its alpha helical coil, which regulated the entire chromosomal passenger complex in mammals [10]. In this study, TAT-CC(T117A) showed the different functions that TAT-CC(T117A) treatment enabled the proportion of cells in G0/G1 phase decreased sharply, and proportion of cells in S phase increased significantly (Figure 5A and 5B). Because Cyclin D1 was first synthesized in cell cycle and peaked at mid-G1 phase [40], the Cyclin D1 level of TAT-CC(T117A)-treated group by western blot was higher than that in TAT-BIR(T34A) group and TAT-Survivin(T34/117A) group (Figure 6). Emerging data supported the notion that TAT-CC(T117A) promoted Bcap-37 cells to leave the G0/G1 resting phase and to enter the stage of DNA replication, and thus promoting cell division. Moreover, we constructed TAT-CC-EGFP to observed localization of recombinant protein in Bcap-37 cells, and demonstrated that CC domain of survivin was able to bind with tubulin, and localize in microtubules (Figure 8B), indicating that the function of CC domain at cell division to control microtubule stability and assembly of a normal mitotic spindle. It was interesting to note that TAT-BIR(T34A) blocked mitosis, while TAT-CC(T117A) promoted process of DNA replication and mitosis.

Autophagy was the two-blade sword that low levels of autophagy were a cytoprotective mechanism, but excessive and continuous autophagy would cause cancer cell death [42]. It was reported YM155, survivin inhibitor induced apoptosis of depended autophagy in prostate cancer cells [43]. Wang et al. [25] also found that suppression of survivin by YM155 induced autophagy-dependent apoptosis, and YM155-induced autophagy played a pro-apoptotic role. According to the result of these previous experiments, we conjectured that up-regulation of survivin inhibited autophagy, whereas down-regulation of survivin promoted autophagy. We mentioned before, single BIR(T34A) was heterologous to wild-type survivin and resulted in the ubiquitin-dependent degradation of survivin, which might be the main cause of survivin to promote autophagy. Based on this idea, we used acridine orange to firstly stain Bcap-37 cells which treated with recombinant proteins. Acridine orange could penetrate into autophagy vacuoles, and emitted red fluorescence in lower pH, and the intensity was related to the degree of acidity [33]. We discovered red fluorescence representing acidic organelle in Bcap-37 cells of TAT-Survivin(T34/117A)- and TAT-CC(T117A)-treated group were dramatically deepened, while fluorescence intensity of TAT-BIR(T34A) group was relatively weak (Figure 9). Therefore, it could speculate that TAT-CC(T117A) might promote autophagy. In addition, LC3B protein was one of microtubule-associated protein 1 light chain 3 (LC3) members characterizing the autophagy, and the levels of LC3B to some extent reflected the number of autophagy body [44, 45]. Immunofluorescence assay discovered intracellular LC3B protein content increased slightly in Bcap-37 cells treated with TAT-BIR(T34A), whereas the levels of LC3B protein increased significantly incubated with TAT-CC(T117A) and TAT-Survivin(T34/117A) (Figure 10), suggesting TAT-CC(T117A) could promote the formation of autophagy vacuoles. Western blot also further yielded similar results, the expression level of LC3B was remarkable higher in Bcap-37 cell incubated with TAT-CC(T117A) than that of other groups (Figure 6). Beclin-1 was a protein that plays a central role in autophagy, and interacted with multiple cofactors (Atg14L, UVRAG, Bif-1, Rubicon, Ambra1, HMGB1, IP3R, PINK, and survivin) to promote the formation of the Beclin-1-Vps34-Vps15 complex which triggered the autophagy protein cascade [46]. The interaction of autophagic governor Beclin-1 and survivin could respond to TRAIL in human glioma cells [24]. The expression of Beclin-1 in TAT-CC(T117A)-treated group was higher than TAT-BIR(T34A)- and TAT-Survivin(T34/117A)-treated group by western blot (Figure 6), suggesting TAT-CC(T117A) could promote autophagy by increased the level of Beclin-1. Therefore, we demonstrated that CC domain of survivin was the main cause of survivin to promote autophagy. The specific mechanism of CC domain of survivin to promote autophagy, however, required further study. Recent research found that Beclin-1 was a direct substrate of caspase-3, caspase-7 and caspase-8 in apoptosis, and the caspase cleavage of Beclin-1 was sufficient to suppress autophagy [23]. TAT-BIR(T34A) and TAT-Survivin(T34/117A) prompted caspase-3 increased and survivin decreased, which caused cleavage of Beclin-1 and inhibited the formation of the Beclin-1-Vps34-Vps15 complex that triggered the autophagy protein cascade. Therefore, BIR domain mutant (BIR(T34A)) slightly promoted autophagy via the caspase-dependent apoptosis, but molecular details of these interactions are poorly understood.

In summary, single BIR domain and CC domain of survivin had showed some functions. Further experiments confirmed that TAT-BIR (T34A) could be used to inhibit cell proliferation, promote apoptosis, and block mitosis, and TAT-CC (T117A) showed mainly to promote autophagy, process of DNA replication, and mitosis to breast cancer cells. Maybe the full-length survivin displayed the functions in the anti-apoptosis, regulating cell cycle, and promoting autophagy by two different domain of survivin. This will lay the foundation for interpreting the multifunction mechanism of survivin in cell fates, and further make senses in the study of cancer pathophysiology, drug discovery, and medical diagnosis targeting survivin precisely and efficiently.

## MATERIALS AND METHODS

### Cell lines and culture conditions

Human breast cell lines Bcap-37 was cultured in Dulbecco’s Modified Eagle Medium (DMEM, Gibco, USA) supplemented with 10% (v/v) fetal bovine serum (FBS, Gibco, USA), 100 μg/mL streptomycin, and 100 unit/mL penicillin. Cells were incubated at 37°C with 5% CO_2_ supply.

### Construction and expression of the recombinant proteins

In order to study the function(s) of each domain of survivin, we used mutant protein TAT-Survivin(T34/117A) (full-length survivin with mutations at T34A and T117A) as a reference. On this basis, we constructed the truncated version of the full-length protein (namely BIR(T34A) and CC(T117A)), fused with penetrating peptide TAT. In addition, the EGFP-tagged proteins were constructed for the purpose of transmembrane efficiency determination and localization.

### Morphology and cell viability assay

Bcap-37 cells in 96-well plate (2×10^4^ cells/well) were divided into two groups, control group and treatment group. The cells in treatment group were incubated with different concentrations (7.5, 15, 30, 60, and 90 µg/mL) of TAT-BIR(T34A), TAT-CC(T117A), and TAT-Survivin(T34/117A), respectively. The control cells were incubated with phosphate buffer solution (PBS buffer). After incubation for 48 h, the supernatant was cleaned away, 180 μL medium and 20 μL MTT (Solarbio, China) solution (5 mg/mL) were added and incubated for 4 h. After incubated for 4 h, MTT-containing medium was removed, and 150 μL DMSO was added to each well to dissolve formazan. The optical densities of the samples were determined by a spectrophotometer (Bio-Tek, USA) at 490 nm. Each experiment was performed independently for three times. Morphologic analysis was performed followed by the treatment of Bcap-37 cells with TAT-BIR(T34A) for 48 h.

### Localization of recombinant proteins and assay of mitochondrial membrane potential

Bcap-37 cells (2 × 10^4^ cells/well) were seeded in confocal petri dish (NEST, China), and incubated at 37°C with 5% CO_2_ supply for overnight. The adhered cells were treated with TAT-CC-EGFP for 48 h. After the supernatant was cleared away, the cells were fixed by 4% (v/v) paraformaldehyde (PA) for 10 min, and then washed three with PBS. Treatment by 0.1% (v/v) Triton X-100 was done at room temperature for 10 min. The cells were blocked for 1 h at room temperature with 3% BSA after cell permeabilization. Incubation with monoclonal antibody against β-tublin was 10 min at room temperature. After the washing steps with PBS, incubation with the secondary anti-β-tublin-cy3 was carried out in the dark for 1 h. Finally, the Bcap-37 cells were washed with PBS, and stained with Hoechst33342 for 10 min in the dark. The cell fluorescence was recorded with a confocal laser scanning microscopy (Nikon, Japan). For mitochondrial membrane potential assay, Bcap-37 cells were treated by TAT-Survivin(T34/117A), TAT-BIR(T34A), and TAT-CC(T117A) for 48 h, and then were stained with Rhodamine 123 by following the instructed protocols.

### Cell cycle and apoptosis assay

Bcap-37 cells (2×10^4^ cells/well) were seeded into 6-well plate (Corning, Elmira, NY) overnight. The cells of apoptosis assay were treated with different concentrations (30, 60 and 90 µg/mL, respectively) of TAT-BIR(T34A) for 36 h or 60 h, then the cells were harvested by centrifugation at 1000 rpm for 8 min. After a twice-wash step with PBS, the cells were re-suspended in 500 µL of Binding Buffer. Staining was done by incubating cells with with 5 µL Annexin V-FITC and 5 µL propidium iodide (PI) (50 µg/mL) (Sangon Biotech, China) for 15 min at room temperature in the dark. The samples were analyzed by flow cytometry assay. The samples for cell cycle assay were prepared by following subsequent procedures: treatment with 30 µg/mL of TAT-BIR(T34A), TAT-CC(T117A), and TAT-Survivin(T34/117A) for 48 h, fixation with ice-cold 70% (v/v) ethanol in PBS (pH7.4) at 4°C overnight after centrifugation at 300 g for 5 min, staining with 50 µg/mL PI ( including 50 μg/mL RNase A) for 30 min. The prepared samples were analyzed by flow cytometry (Becton Dickinson, USA).

### Autophagy analysis

Bcap-37 cells (2 × 10^4^ cells/well) were seeded into 6-well plate overnight and then treated with TAT-BIR(T34A), TAT-CC(T117A), and TAT-Survivin(T34/117A) for 48 h. The harvested cells were stained for 15 min with 0.15 μmol/L acridine orange (AO) and washed three times with PBS. The fluorescence was observed with microscopy (OLYPAS, Japan).

### Western blot analysis

Cells were plated onto 6-well plate at a density of 2×10^4^ cells/well. The adhered cells were treated by TAT-BIR(T34A), TAT-CC(T117A), or TAT-Survivin(T34/117A) at the concentration of 60 µg/mL for 48 h and washed twice with PBS. The cells were harvested by centrifugation at 1000 rpm for 8 min and washed once with ice-cold PBS, and then lysed in 200 μL RIPA buffer for 30 min on ice with gentle rocking. The total protein concentration was determined by the BCA protein assay method with BSA as standard. Proteins (50 μg) were loaded into each lane and separated by 15% SDS-PAGE, then were electro-transferred onto pretreated PVDF membranes. The membranes were blocked for 2 h at room temperature with 5% non-fat milk in PBS containing 0.05% Tween-20 (PBST). Then was probed with antibodies against activated caspase-3 (CST, USA), activated caspase-9 (CST, USA), survivin (CST, USA), Cyclin D1 (CST, USA), Beclin-1 (CST, USA), LC3B (CST, USA), and β-actin (Sangon Biotech, China) for 1 h at room temperature. After washed with PBST for three times, and the membrane was incubated with the secondary mouse anti-rabbit IgG-HRP for 1 h at room temperature. Protein bands were visualized on X-ray film with enhanced ECL Western blotting detection reagents (Sangon Biotech, China). The grey values of protein bands have been quantified and statistically analyzed by Image J.

### Statistical analysis

SPSS software version 22.0 was used for the statistical analysis of the data. Data were expressed as the mean ± SD based on the results obtained from at least three independent experiments. A level of *P* less than 0.05 was considered to be statistically significant.
